# Review: Posed vs. Genuine Facial Emotion Recognition and Expression in Autism and Implications for Intervention

**DOI:** 10.3389/fpsyg.2021.653112

**Published:** 2021-07-09

**Authors:** Paula J. Webster, Shuo Wang, Xin Li

**Affiliations:** ^1^Department of Chemical and Biomedical Engineering, Rockefeller Neuroscience Institute, West Virginia University, Morgantown, WV, United States; ^2^Lane Department of Computer Science and Electrical Engineering, West Virginia University, Morgantown, WV, United States

**Keywords:** facial expression of emotion, emotion recognition, posed vs. genuine emotion, autism spectrum disorder, social deficits

## Abstract

Different styles of social interaction are one of the core characteristics of autism spectrum disorder (ASD). Social differences among individuals with ASD often include difficulty in discerning the emotions of neurotypical people based on their facial expressions. This review first covers the rich body of literature studying differences in facial emotion recognition (FER) in those with ASD, including behavioral studies and neurological findings. In particular, we highlight subtle emotion recognition and various factors related to inconsistent findings in behavioral studies of FER in ASD. Then, we discuss the dual problem of FER – namely facial emotion expression (FEE) or the production of facial expressions of emotion. Despite being less studied, social interaction involves both the ability to recognize emotions and to produce appropriate facial expressions. How others perceive facial expressions of emotion in those with ASD has remained an under-researched area. Finally, we propose a method for teaching FER [FER teaching hierarchy (FERTH)] based on recent research investigating FER in ASD, considering the use of posed vs. genuine emotions and static vs. dynamic stimuli. We also propose two possible teaching approaches: (1) a standard method of teaching progressively from simple drawings and cartoon characters to more complex audio-visual video clips of genuine human expressions of emotion with context clues or (2) teaching in a field of images that includes posed and genuine emotions to improve generalizability before progressing to more complex audio-visual stimuli. Lastly, we advocate for autism interventionists to use FER stimuli developed primarily for research purposes to facilitate the incorporation of well-controlled stimuli to teach FER and bridge the gap between intervention and research in this area.

## Introduction

Individuals with autism spectrum disorder (ASD) often have difficulty interpreting and regulating their own emotions, understanding the emotions expressed by others, and labeling emotions based on viewing the faces of others (Harms et al., 2010; Uljarevic and Hamilton, 2013; Sheppard et al., 2016). These differences can contribute to social self-isolation by those with ASD either when others respond negatively if the person with ASD lacks a typical, socially expected response, or if the person with ASD chooses to socially isolate themselves to avoid possibly stressful interactions if they realize they struggle to recognize and respond appropriately to expressions of emotion by others (Jaswal and Akhtar, 2019).

Research investigating facial emotion recognition (FER) in ASD has primarily utilized static images composed of posed facial expressions (Pelphrey et al., 2007; Monk et al., 2010); however, more recent research has begun exploring the use of dynamic video with actors making posed facial expressions (Golan et al., 2015; Fridenson-Hayo et al., 2016; Simões et al., 2018). Few studies have utilized face stimuli of humans expressing genuine, spontaneous expressions of emotion, whether static or dynamic (Cassidy et al., 2014). This distinction is important because research has shown that the human brain, and artificial intelligence (AI) systems, process posed facial expressions differently compared to how spontaneous expressions of emotion are processed (Hess et al., 1989; Schmidt et al., 2006; Wang et al., 2015; Park et al., 2020).

Results have been mixed with most studies indicating that posed expressions of emotion being easier to recognize than those that are spontaneous (Naab and Russell, 2007); however, accuracy for FER may also depend on the specific emotion being evaluated (Faso et al., 2014; Sauter and Fischer, 2018). This may be due to the prototypical nature of posed expressions (e.g., most people show fewer teeth when they smile for posed pictures; Van Der Geld et al., 2008), whereas there is much more variability in genuine expressions of some feelings such as sadness (Krumhuber et al., 2019). Therefore, it has been proposed that the traditional use of posed facial expression stimuli in research may have artificially inflated behavioral measures of accuracy during emotion recognition tasks (Sauter and Fischer, 2018). Therefore, the historically prevalent use of posed facial expression stimuli in ASD research investigating FER may contribute to the mixed results seen in this research area.

How might these dissimilarities in posed vs. spontaneous facial expression stimuli be perceived differently by those with ASD? This review further argues that posed vs. genuine emotion is a critical factor that deserves more consideration when studying FER in ASD. We will first review the rich literature on the perception of posed facial expressions of emotion, highlighting the differences between ASD and control groups, though inconclusively. We will then discuss some recent research investigating how individuals with ASD differ from controls when asked to produce posed facial expressions of emotion and review the latest advances in the field of posed vs. spontaneous/genuine facial expressions and implications into autism research in terms of both perception and production of genuine facial expressions. Finally, based on these findings, we propose a method of teaching FER for individuals with ASD.

## Differences in FER in ASD

Autism studies investigating differences in understanding how others think or feel date back to as early as the 1970s (Langdell, 1978; Mesibov, 1984; Weeks and Hobson, 1987; Hobson et al., 1988; Ozonoff et al., 1990). In Langdell (1978) they found that adolescents with autism could identify schematically drawn happy and sad faces, but they demonstrated varying capability when sorting the faces just using the eye area. Another study (Hobson, 1986) provided further convincing evidence about the differences in the appraisal of facial expressions of emotion by children with autism suggesting that their failure to understand the emotional states of others might be related to their difficulty in recognizing the difference between particular emotions. However, due to different experimental designs (e.g., sorting, matching, and cross-modal), the interpretation of these early results is often debatable (Celani et al., 1999).

A more systematic study about the nature of early differences in social cognition in autism was conducted in Dawson et al. (2004) using high-density event-related potentials (ERPs). It was found that children with ASD, as young as 3 years of age, showed a disordered pattern of neural responses to emotional stimuli such as fearful vs. neutral facial expressions. More specifically, typically developing children demonstrated a larger early negative component and a negative slow wave to the fear than to the neutral, while children with autism did not show significant differences in both experiments. In contrast, the faster speed of early processing of the fear face among children with autism was associated with better performance on tasks assessing social attention such as social orienting, joint attention, and attention to distress. These findings have served as direct evidence for atypical psychological components involving emotion recognition among children with autism at a young age (3–4 years old).

To probe into the pathology of the underlying processes related to dysfunction in emotional and social cognition, it has been shown that amygdala dysfunction in ASD might contribute to a different ability to process social information (Adolphs et al., 2001). Varying face perception or emotion recognition in ASD might result from atypical fixations onto faces, which may, in turn, arise from amygdala dysfunction (Breiter et al., 1996; Baron-Cohen et al., 2000). This hypothesis is directly supported by evidence from both single-neuron recordings in the human amygdala (Rutishauser et al., 2013) and neuroimaging studies (Dalton et al., 2005; Kliemann et al., 2012). Given the critical role of the amygdala in emotion processing (Adolphs, 2008), more systematic studies will be needed to reveal whether the amygdala has a different response for posed vs. genuine emotions. Further studies using visual scanning/eye-tracking (Pelphrey et al., 2002), or functional neuroimaging (Dalton et al., 2005; Pelphrey et al., 2005), have shown abnormal activity in patients with ASD. Even with the enhanced emotional salience of facial stimuli, a positron emission tomography (PET) study showed that adults with ASD demonstrated lower activity in the fusiform cortex than typically developing (TD) controls and differed from the TD group within other brain regions (Hall et al., 2003). This line of research was further extended into the identification of differences in key components of human face processing systems that might contribute to the differences in processing facial expressions of emotion (Pelphrey and Carter, 2008).

Unlike previous studies employing more simplistic stimuli (e.g., the face stimulus as an exemplar of a given emotion, “100% expression”), subtle differences in FER were considered (Law Smith et al., 2010; Black et al., 2020). Using stimuli that incrementally morphed the expression between a neutral face and the posed expression, they found that adolescents and young adults with ASD were less accurate at identifying basic emotional expressions of disgust, anger, and surprise. In a follow-up study (Kennedy and Adolphs, 2013), adults with ASD were found to give ratings that were significantly less sensitive to a given emotion and less reliable across repeated testing. Therefore, an overall decreased specificity in emotion perception suggests a subtle but specific pattern of differences in facial emotion perception among those with ASD. Along this line of research, significant differences were found between males and females with ASD for emotion recognition but not for self-reported empathy recognition (Sucksmith et al., 2013). Most recently, a gender-biased study showed that differences in FER in *females* with autism might not be attributed to ASD but instead to their co-occurring alexithymia (difficulty describing one’s own emotions and those of others; Ola and Gullon-Scott, 2020). Thus, consideration for future FER studies is to recruit significant numbers of male and female participants with ASD and consider sex as a factor in the analysis.

We note that there have been several excellent review articles about research findings of FER in ASD (Harms et al., 2010; Bons et al., 2011; Nuske et al., 2013; Uljarevic and Hamilton, 2013). In Harms et al. (2010), demographic and experiment-related factors are addressed to account for inconsistent findings in behavioral studies of FER in ASD. Future studies of FER in ASD suggested by Harms et al. (2010) include the incorporation of longitudinal designs to examine the developmental trajectory of FER and behavioral and brain imaging paradigms that include young children. In Uljarevic and Hamilton (2013), a formal meta-analytic study has shown that recognition of happiness was only marginally modified in ASD, but recognition of fear was marginally worse than recognition of happiness. In Nuske et al. (2013), it was found that (1) emotion-processing differences might not be *universal* to all individuals with ASD and are not *specific* to ASD; and (2) the specific pattern of emotion-processing strengths and weaknesses observed in ASD, involving difficulties with processing social vs. nonsocial, and complex versus simple emotional information, appears to be *unique* to ASD (Tang et al., 2019). It is also worth noting the “double empathy problem” described (Milton, 2012). It was found that just like people with ASD have difficulty interpreting the facial emotions of TDs, TD people have just as much difficulty understanding people with autism. Such a “double” perspective has profound implications for ASD service providers because differences in neurology could lead to differences in sociality. A more recent study (Milton and Sims, 2016) has demonstrated a need for less focus on remediation for patients with autism. Instead, it advocated for focusing on limiting social isolation as a more constructive solution. The most recent study (Crompton et al., 2020) has shown that peer-to-peer information transfer concerning autism is more effective than information transfer between persons with and without autism.

Given the finding that FER differences are not strictly applicable to those with ASD (Nuske et al., 2013), several studies have been conducted to compare differences in FER in ASD with other neurological disorders. In Wong et al. (2012), emotion recognition abilities are examined for three groups of children aged 7–13 years: high functioning autism (HFA), social phobia (SP), and TD. Although no evidence was found for negative interpretation biases in children with HFA or SP, children with HFA were found to detect mild affective expressions less accurately than TD peers suggesting subtle changes in emotion expression are more difficult for those with ASD. In Sachse et al. (2014), a similar study was conducted with adolescents and adults with HFA, schizophrenia (SZ), and TD to identify convergent and divergent mechanisms between ASD and SZ. It was found that individuals with SZ were comparable to TD in all emotion recognition measures, but the basic visuoperceptual abilities of the SZ individuals were reduced. By contrast, the HFA group was more affected in recognizing basic and complex emotions when compared to both SZ and TD. As reported in Sachse et al. (2014), group differences between SZ and ASD remained but only for recognizing complex emotions after taking facial identity recognition into account. Such experimental results suggest that (1) there is an SZ subgroup with predominantly paranoid symptoms that do not show problems in FER but visuoperceptual differences only; and (2) no shared FER difference was found for paranoid SZ and ASD, implying differential cognitive underpinnings of ASD and SZ about FER.

A study by Lundqvist (2015) directly links sensory abnormality with social dysfunction of ASD – for example, hyper-responsiveness to touch mediated social dysfunction in ASD, and the tactile sensory system is foundational for social functioning in ASD. There is also evidence that social functioning in those with ASD is impacted by sensory dysregulation in multiple sensory modalities that arise early in the progression of the disorder (Thye et al., 2018). This meta-analysis suggests an early intervention that targets sensory abnormalities and social differences, considering the critical role ASD sensory processing differences play in social interactions. In another systematic review and meta-analysis (Zhou et al., 2018), quantitative comparisons of sensory temporal acuity were made between healthy controls and two clinical groups (ASD and SZ). They revealed a consistent difference in multisensory temporal integration in ASD and SZ, which may be associated with differences in social communication. Finally, studying differential patterns of visual sensory alternation using neuroimaging (Martínez et al., 2019) has shown that SZ and ASD participants demonstrated similar FER and motion sensitivity differences, but significantly different visual processing contributed to FER group differences. This data would suggest that FER differences are not unique to ASD.

## Differences in Facial Emotion Expression in ASD

It has been hypothesized that in ASD, both FER and FEE are affected, contributing importantly to social differences and difficulty in relationship formation (Manfredonia et al., 2019). By contrast, fewer studies about individuals with ASD have been devoted to FEE than FER in the published literature. In an early study of imitation and expression of facial affect (Loveland et al., 1994), the production of elicited/posed affective expressions is more difficult for individuals with ASD than for patients with Down’s syndrome of similar chronological age, mental age, and IQ. In Begeer et al. (2008), four aspects of emotional competence (expression, perception, responding, and understanding) are reviewed for children and adolescents with ASD. It was found that different emotional competence in ASD was highly dependent on age, context, and intelligence. In another unique study (Faso et al., 2014), the dual problem of FER and FEE were studied, namely how facial expressivity by those with ASD is perceived by others. It was reported that facial expressions of emotion by participants with ASD were regarded as more intense and less natural than expressions by the TD group. Surprisingly, ASD expressions were also identified with greater accuracy by TD judges due primarily to the category of angry expressions. The above findings collectively suggest differences, instead of a reduced ability, in facial expressivity among individuals with ASD. Those differences do not necessarily hinder the accuracy of emotion recognition by others but may affect the quality of social interactions between ASD and TD, as demonstrated in a recent study (Sasson et al., 2017).

In Volker et al. (2009), each participant was photographed after being prompted to enact a facial expression from one of six basic emotions – happiness, sadness, anger, fear, surprise, and disgust. It was reported that children with HFA were significantly less adept at enacting sadness, and their expressions were dramatically odder compared to controls. However, no significant differences were found for anger and fear; and even more surprisingly, the ASD group demonstrated somewhat greater skills at enacting surprise and disgust. More recently, a systematic study (Brewer et al., 2016) investigated TD and ASD participants’ ability to recognize facial expressions of emotion produced by TD and ASD actors posing basic emotions. With three designed posing conditions, this study aimed to determine whether potential group differences were due to (1) atypical cognitive representations of emotion; (2) affected the understanding of the communicative value of expressions; or (3) poor proprioceptive feedback. They found that expressions posed by participants with ASD were not recognized as well by TD and ASD participants as expressions posed by TD posers. Subsequently, a computational approach was used in Guha et al. (2018) to study the details of facial expressions for children with HFA. This study aimed to uncover subtle characteristics of facial expressions by analyzing localized facial dynamics and found differences in the eye region. Finally, in a meta-analysis (Trevisan et al., 2018), it was found that participants with ASD display facial expressions less frequently and for less amount time. Meanwhile, participants with ASD are less likely to share facial expressions with others or automatically mimic the expressions. These observations have partially inspired the design of an intervention system for young children with ASD, as we will elaborate later.

## Posed Vs. Genuine Facial Expressions of Emotion

Multiple databases of face stimuli have been developed for FER research (Jia et al., 2020). These databases include static images of computer-generated human faces that can be titrated to modify facial expressions or include static or dynamic images of real human faces containing posed and spontaneous facial expressions (Cassidy et al., 2015). More recently, there has arisen a question in the emotion recognition field regarding whether there is a difference between how the human brain perceives and processes emotions that are posed (artificially generated) compared to those that are genuine (spontaneously generated). One study found that adults are much more accurate at labeling emotions when the facial expression is posed than when it is spontaneous (Krumhuber et al., 2019). In this study, they also used facial recognition software to label the emotions and found the software to be more accurate than the human participants at FER for the posed emotions; however, the accuracy dropped for AI and the human participants to similar levels when the expressions of emotion were spontaneous. It was thought that this result was due to the fact that posed expressions showed more prototypical facial features of the emotions (e.g., downturned mouth and furled brow for sadness) enabling both humans and AI to learn and recognize the posed emotions with higher accuracy. Spontaneous emotional expressions have subtle, but substantial differences compared to posed expressions of emotion, with changes in small muscles and less prototypical facial expressions (Kim and Huynh, 2017). Few studies have compared FER for posed and genuine FEs with mixed results. Here, we will first highlight a few existing studies on posed vs. genuine facial expressions of emotion for ASD and then discuss our envisioned future directions along this line of research.

Recent studies had revealed differences in the literature when processing posed vs. genuine facial expressions of emotion (Pelphrey et al., 2007). There are prototypical signs exhibited for some expressions of emotion, while genuine expressions of the same emotion are more complex and harder to interpret. For example, the expression of sadness when posed includes an out-turned lower lip, though spontaneous expressions of sadness are much more highly variable and often do not include this prototypical expression (Kim and Huynh, 2017). The class of smile expressions has received special attention regarding posed vs. genuine distinction (Blampied, 2008; Boraston et al., 2008). In Blampied (2008), the sensitivity of children with ASD was compared against that of age and sex-matched control children to the different emotions underlying posed vs. genuine smiles. It was found that individuals with ASD are often less sensitive to the differences between posed and genuine smiles than TD participants. Toward deeper reasoning about this difference, it was hypothesized that experience during development viewing the eye region of a face is critical to identifying genuine smiles from posed ones. In a related study (Boraston et al., 2008), the reduced ability to discriminate genuine from posed smiles for adults with ASD is attributed to reduced eye contact. It was also found that the individuals with ASD who were more affected in recognition of genuine smiles also had more severe social interaction differences. In a recent review of studies using eye-tracking (ET) and electroencephalography (EEG) to explore FER in ASD (Black et al., 2017), they report that differences in ET and EEG result from differences in facial emotion processing that arise from functional differences in the social brain.

Evaluating posed and evoked facial expressions of emotion from adults with ASD has been studied (Faso et al., 2014). It was reported that ASD expressions were rated as more intense and less natural than TD expressions. Meanwhile, the naturalness ratings of evoked expressions were positively associated with identification accuracy for TD but not individuals with ASD. These findings collectively highlight differences in facial expressivity among ASD that do not hinder emotion recognition accuracy but may affect the quality of social interaction. Along this line of research, it has also been found that just like the failure of ASD recognize the facial expressions of TD (no matter posed or spontaneous), TD individuals also find it difficult to recognize autistic emotional expressions (Brewer et al., 2016). More recently, it has been found that neurotypical peers are less willing to interact with those with autism based on thin slice judgments (Sasson et al., 2017), and first impressions for intellectually able adults with ASD improve with diagnostic disclosure and increased autism understanding of the part of peers (Sasson and Morrison, 2019).

Considering the differences in TD accuracy for posed and spontaneous FEs, it would stand to reason that differentiating these types of stimuli in autism interventions targeting FER should be considered. Next, we propose a progressive intervention strategy inspired by research investigating posed vs. genuine expressions of emotion.

## Implications for ASD Intervention

While FER differences in individuals with ASD may not be universal, they are highly prevalent, and thus FER is often specifically taught as part of the autism curriculum of a child (Ayres and Robbins, 2005). Interventions have been developed that explicitly teach individuals with ASD to recognize specific emotions in others and themselves with mixed results (for a review, see Berggren et al., 2018). Stimuli for FER interventions can vary widely and may include static or dynamic images of the six basic emotions (i.e., sad, happy, angry, afraid, disgust, and surprise) as well as complex emotions, such as jealousy, that are more difficult to recognize and may require the use of contextual clues (Baron-Cohen et al., 2009). The basic goal of teaching FER to those with ASD is to help them better understand others and foster communication and social interactions (core difference areas in ASD). Previous works (Gordon et al., 2014) have focused on how to train children with ASD to produce happy and anger expressions with a computer game (“FaceMaze”). Recently, technology-based learning tools have been designed to help ASD preschoolers with FER and emotional understanding (Boccanfuso et al., 2016; Zhang et al., 2019).

Additionally, based on the observation that happiness is the easiest among the six basic emotions for encoding and decoding by humans, a computer-based tutoring system called *SmileMaze* (Cockburn et al., 2008) was designed to improve the FEE production skills of children with ASD in a dynamic and interactive format. The Computer Expression Recognition Toolbox (CERT) in *SmileMaze* is capable of automatically detecting frontal faces from a video stream and encoding each frame into 37 continuous features, including six basic facial expressions as well as 30 facial action units (AUs) as defined by the Facial Action Coding System (Ekman, 1997). Such a computational approach notably targets those characteristics in ASD that are distinct from those in TD children, which are often difficult to detect by direct visual inspection. The combination of FEE training and computer vision systems leads to the most recent work (White et al., 2018) – an automated, game-like system based on the Kinect 3D sensing technology developed by Microsoft. It has been reported that youth with ASD preferred to interact with the system more than their TD peers. Such a discovery seems to suggest that new technology-based interventions (e.g., 3D avatar-based digital twin; Wang et al., 2019), music-based therapeutic methods (Wagener et al., 2020), and computer-based recognition of posed vs. spontaneous facial expressions (Mavadati et al., 2016), have good potential in remediation of transdiagnostic processes such as FER and FEE in ASD and possibly in other disorders with facial emotion processing differences such as SZ, traumatic brain injury, and stroke. It has recently been reported in Keating and Cook (2021) that individuals with autism have difficulties recognizing neurotypical facial expressions and vice versa. TD and ASD individuals might exhibit expressive differences, but individuals with autism tend to display less frequent expressions that are rated as lower in quality by TD observers. Such observation suggests that future research should investigate what specifically is different about the facial expressions produced by ASD and TD individuals (e.g., how dynamic aspects of expressions affect emotion recognition).

Considering the scientific literature outlined in this review on FER in ASD and differences between posed and genuine facial expressions of emotion discussed above, we propose a hierarchical teaching method as part of an intervention to teach FER to individuals with ASD that considers the increased difficulty in processing more complex FER stimuli (Nuske et al., 2013). We propose three aspects for consideration when teaching FER: (1) whether the image is simple (drawings and cartoons) or complex (includes human faces or life-like artificially generated faces); (2) whether the image is static or dynamic [audio-visual (AV)]; and (3) in complex images, whether the expression of emotion is posed or genuine. Those three aspects collectively take previous findings in the literature of FER/FEE in ASD into consideration and introduce a new sequential approach toward posed vs. genuine. Compared with previous approaches such as SmileMaze (Cockburn et al., 2008) and FaceMaze (Gordon et al., 2014), ours distinguishes them by emphasizing hierarchical learning and covering more facial expressions.

We propose two possible approaches for teaching FER/FEE:

**Approach (1) Teaching FER/FEE Progressively**: This strategy is based on the previous finding that happiness and sadness are the least affected in ASD, but fear, surprise, and disgust are more impacted in ASD. Starting with simple, static images that include basic drawings and cartoon characters and then progressing step-wise to more complex static images with photos of human faces and expressions that are posed and genuine, and then to dynamic AV images using a life-like avatar of the therapist or child conversing in real-time as a transition between static and dynamic images of real people, and finally, real-world AV videos that contain context clues and genuine expressions of emotion. While using photos of real faces constitutes a more natural stimulus and may positively impact generalizability, the simplicity of the hand-drawn images may make them a better place to begin teaching emotions for some individuals with ASD (Sasson et al., 2008). In this vein, similar to standard Applied Behavior Analysis (ABA) methods, once they have mastered an emotion at the hand-drawn image level, it may be beneficial to move to the next level of complexity and target cartoon characters that the child enjoys. Theoretically, intervention would then move to the inclusion of stimuli with real human faces with posed emotions because posed photos are easier for typically developed individuals to label. Ultimately, using real human face stimuli with genuine, spontaneous expressions of emotion (static or dynamic) would be the ultimate target since they may be more difficult to interpret (Hanley et al., 2013). Images of the child undergoing intervention that shows him/her expressing these emotions could also be included and analysis of their facial expressions.

**Approach (2) Teaching FER/FEE in a Field of Images**: Alternatively, since individuals with ASD often have difficulty generalizing what they have learned in many areas, including FER (Berggren et al., 2018) and FEE (White et al., 2018), it may be best, to begin with, multiple images of a specific emotion to teach a child (e.g., in a field of drawn images, cartoon characters, and posed and random static photos of human faces expressing a target emotion). Teaching skills to individuals with ASD in a field of stimuli has been proposed previously based on the finding that repeatedly using limited stimuli increases the rigidity of thinking and reduces generalizability (Harris et al., 2015). Thus, in Approach 2, we propose to begin by teaching FER in a field that contains static images that are both simple and complex of posed and genuine expressions of a target emotion and then progress to dynamic AV FER stimuli that may contain more context clues and incorporates multisensory integration to facilitate learning (Sasson, 2006). While teaching in a field may take longer to master, research shows it may reduce learned rigidity of thought and improve generalizability. Finally, incorporating these stimuli into games that are enjoyable to play (see above referenced FER/FEE interventions), and could be customized so that the interventionist can select the images at each level of FER/FEE functioning, could facilitate facial emotion training in some individuals with ASD.

While the intrinsic social motivations of a child may not significantly impact how FER/FEE is taught (Garman et al., 2016), delivering the stimuli in a fun and intrinsically motivating way could improve generalizability (Baron-Cohen et al., 2009). A feasibility study was conducted by White et al. (2018) of their system developed to teach FEE to children with ASD. The system provides critical feedback to the child *via* computer analysis of the facial expression a child made in response to a cue. Such a system could be used in conjunction with a FER/FEE training program since the ability of a child to recognize their own emotions may likely facilitate FER/FEE learning and thus may be a framework upon which recognition of others’ emotions can be built (Manfredonia et al., 2019; Ola and Gullon-Scott, 2020). Additionally, avatars can be created to interact in real-time with a child and may provide an added opportunity for a person with ASD to initiate conversations of their own accord, as has been seen at Disney World where children with ASD willingly interact with an avatar of Crush the turtle from the movie *Nemo* (Carter et al., 2014). Regarding the dynamic AV stimuli, since multisensory integration has been shown to enhance our ability to learn new information (Shams and Seitz, 2008), incorporating auditory input with visual input may facilitate the ability for individuals with ASD to learn emotion recognition, especially at the more complex levels of FER/FEE as in, where the stimuli would be considered the most complex (real-world, AV, and genuine expressions of emotion). Consideration should be given to the level of functioning of an individual in face/emotion processing and learning style when determining where to begin and whether to teach progressively (Approach 1;) or to teach in a field (Approach 2) of static images and then progress to dynamic AV videos.

Additionally, the scientific community has developed multiple datasets of face stimuli for research purposes to investigate how FER/FEE is perceived in TD, ASD, and other disorders (for a review of FER databases, see Jia et al., 2020). These stimuli have static and dynamic expressions of emotion that are often well titrated (morphed levels between two emotions), but these stimuli are generally not known to autism therapists and are not utilized by them for teaching FER/FEE. Thus, the availability of face stimuli for teaching is often dependent upon the funds available to an interventionist. Therapists have been very creative and find free face stimuli to use when teaching their students, which can benefit children when various images are used. However, this can be time-consuming and costly, especially if therapists must purchase images from different datasets to acquire a set of images for teaching a specific emotion. Therefore, we propose that interventionists take advantage of the variety of FER datasets that include both posed and genuine expressions of emotion and dynamic videos of facial expressions of emotions.

Many FER/FEE databases have been developed using the six basic emotions that were found to be universal (Ekman, 1970) and the Face Action Coding System (FACS) that breaks down movements of muscles in the face used to make expressions of emotion into AUs (Ekman, 1997). These same AUs are the primary measures for facial expressions used by entities like Disney to animate characters to make their facial movements more realistic. Thus, using stimuli to teach FER/FEE in those with ASD that has incorporated realistic portrayals of human emotions (e.g., Disney characters) and analyses of human expressions of emotion based on these same measurements of facial micromovements (Leo et al., 2019) would bring full circle the application of the research investigating this critical aspect of human existence to help those who struggle in this area. Lastly, avatar software developed by companies like ObEN can benefit FER intervention by enabling the creation of life-like avatars of a therapist or of the person with ASD,^1^ which may help individuals with ASD to transition between static images and dynamic real-world videos that contain context clues and possibly help them better understand their own expressions of emotion.

The proposed FETH method requires research to investigate the merits of teaching FER/FEE serially (Approach 1) or teaching in a field of images at different levels of complexity to improve generalizability (Approach 2). Regardless, a more refined FER/FEE intervention based on current scientific outcomes has far-reaching implications for children and adults with ASD and other disorders where FER/FEE difficulties can significantly hinder social interactions, including SZ, stroke, and traumatic brain injury.

## Author Contributions

PW, SW, and XL contributed to the research, analysis, and writing of the manuscript. All authors contributed to the article and approved the submitted version.

### Conflict of Interest

The authors declare that the research was conducted in the absence of any commercial or financial relationships that could be construed as a potential conflict of interest.

## Footnotes

^1^https://oben.me
